# Water Quality and Pollution Trading: A Sustainable Solution for Future Food Production

**DOI:** 10.1021/acsestengg.2c00383

**Published:** 2023-07-13

**Authors:** Jamie Gonzalez Zapata, Bharadwaj Vangipuram, Carole Dalin, Tohid Erfani

**Affiliations:** † Department of Civil, Environmental and Geomatic Engineering, University College London, London WC1E 6BT, U.K.; ‡ Institute for Sustainable Resources, Bartlett School of Environment, Energy and Resources, University College London, London WC1H 0NN, U.K.

**Keywords:** water quality trading, nitrogen pollution, agriculture, food security, market-based model

## Abstract

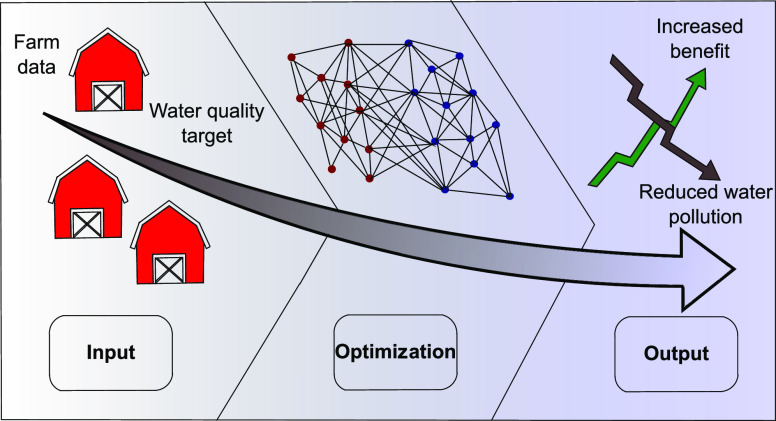

Nitrogen, an essential nutrient for plant growth, is commonly added to food crops in the form of manure and synthetic fertilizers. Fertilizer use has significantly increased in the past decades to meet the food demands from a rising population. Although this has boosted food production, it has come at a cost to the environment. Indeed, excess fertilizer ends up in water bodies, a pollution that causes losses in aquatic biodiversity. Better fertilizer management is therefore essential to maintaining water sustainability. Here, we develop and evaluate a nitrogen water quality trading scheme to address this challenge. Nitrogen trading incentivizes farmers to work together to invest in pollution reduction measures in order to keep nitrogen water pollution levels within a standardized limit. We build a mathematical model to represent the nitrogen trading and use it to assess the pollution reduction, the effect on the crop yield, and economical outcomes. The model is applied among local farms in the agricultural county of Suffolk, eastern England. We calculate the nitrogen load to the river from each farm and incorporate the abatement cost into the model. The results show how nitrogen water pollution could be reduced cost-effectively while simultaneously increasing the benefit for the whole catchment. Although the benefit does not increase for all the farms, the increase in benefit for the whole catchment is enough to compensate for this loss. The surplus benefit is equally distributed between all the farms, thus increasing their overall benefit. We discuss how the proposed trading model can create a platform for farmers to participate and reduce their water pollution.

## Introduction and Background

Population growth and an increase in demand for resource-intensive foods such as meat have increased pressure on the agriculture sector.^1,2^ Nitrogen, an essential element for building proteins and amino acids, is a crucial nutrient for plant growth and the plant life cycle.^3,4^ Nitrogen in its reduced form is scarce in the environment, and therefore food production cannot rely on the nitrogen cycle alone; therefore, the availability of nitrogen is central to food security. However, an increase in nitrogen application use in agriculture to meet current food demands has led to an increase in nitrogen being lost to surrounding water bodies through different pathways—such as leaching, erosion, and surface runoff—thus polluting the environment.^5,6^ The accumulation of nutrients in waterbodies, such as lakes and rivers, particularly the accumulation of nitrogen is leading to nutrient over enrichment and is one of the leading causes of water impairment.^7^ This has an impact on both the ecosystem due to the lowering of water’s oxygen levels, changing the chemical composition of water,^8^ and on human health, as drinking water with high levels of nitrogen has adverse effects on health.^9^

In the past, the main sources of water pollution were sewage and industrial discharges (point sources).^5^ However, as these sources have become more regulated and controlled through treatment and disposal technologies, agriculture, a non-point source, is now the leading cause of water pollution, even in the developed world.^5^ To tackle water impairment from agriculture, much attention and research has been devoted to better managing nitrogen use locally, hence increasing nitrogen use efficiency (NUE)^10^ In addition, some government agencies are implementing nutrient caps coupled with Water Quality Trading (WQT) schemes—for example, the Long Island Sound in the US and Lake Taupo in New Zealand.^11−14^ Nutrient caps put a limit on pollution discharges and can be applied to individual polluting sources or to larger geographic areas, such as a watershed.^15−17^ Reducing nutrient discharges can be costly,^18^ therefore, WQT is used as a means of reducing costs by allowing those who can reduce their discharges most cost-effectively to do so and to sell their nutrient reduction to those with higher nutrient reduction costs.^19−21^ WQT provides the ability to shift nutrient emissions from a low economic value use to a higher economic value use, thus providing flexibility in land-use decisions.^22,23^

Countries with existing WQT are Australia, Canada, New Zealand, and the US. Successful WQT systems, such as the Long Island Sound Nitrogen Credit Exchange program and the Great Miami Trading Program (GMTP), have helped reduce water impairment.^12,13,19^ However, as with most WQT, concerns arise when allocating and tracing nutrient discharges to and from non-point sources because of their diffuse pathways.^24^ Non-point sources emit pollution in a stochastic manner; therefore, their emissions differ spatially and over time.^25^ On top of this, measuring non-point source discharges accurately is challenging, and therefore, non-point sources are not as regulated as point sources. The Long Island Sound program found that setting a cap on pollution emissions can subsequently decrease the land value and may drive farmers away.^26,27^ The WQT system will ensure that water quality standards are met at all times while simultaneously lowering the nitrogen load, reducing associated costs, and maximizing the farms’ economic benefit.^28−31^

This paper contributes to the evolving literature on WQT programs applied at a catchment scale by introducing a simulation market-based model that addresses specifically non-point sources of pollution and specifically with a focus on farms. The WQT model tracks the nitrogen load from the pollution source (farm) to the receiver (surface water) in a pairwise trade applicable at a catchment scale. In the proposed WQT model, all the farms in the catchment must work together to reduce the cost associated with pollution reduction. In addition, we quantify the nitrogen load contribution from each farm using empirical data, and we quantify the impact this nitrogen loading has on nitrogen water pollution. On top of observing trading patterns, we also look at food sustainability and the predicted crop yield changes based on the nitrogen load reductions. To our knowledge, WQT has yet to be applied to a UK river. We demonstrate how a market-based system will function in a real-life scenario by applying this model to the River Alde in Suffolk. We explore how this WQT can reduce nitrogen water pollution cost-effectively with the above in mind.

## Method

### Proposed Approach

We use a network-based mathematical model to develop the WQT model. The proposed model uses a network of nodes (farms) and their links (water pathways) that allows traceability from the polluting source (farm) to the receiving water body.^32^ This allows for all the possible flow pathways to be predefined; therefore, the owner of the nitrogen load (farm) can be linked to a market of buyers and sellers. In this model, each non-point pollution source (farm) will be given a limit on how much nitrogen load they can pollute for one crop growing period, and this will be their nitrogen water pollution license. The summation of the non-point sources (farms) nitrogen water pollution licenses will be the nitrogen water pollution cap set at the waterbody gauge. We use an optimization-based model wherein the sum of the net benefits for all the farms in the model is maximized, and the cost of reducing pollution is reduced at each time step.^33^ In addition, constraints are added to the model to create a real-life trading scenario. This paper focuses on the nitrogen load contribution from farms to surface water. The main aspects of the model are summarized below, followed by the main assumptions.

**Figure 1:**
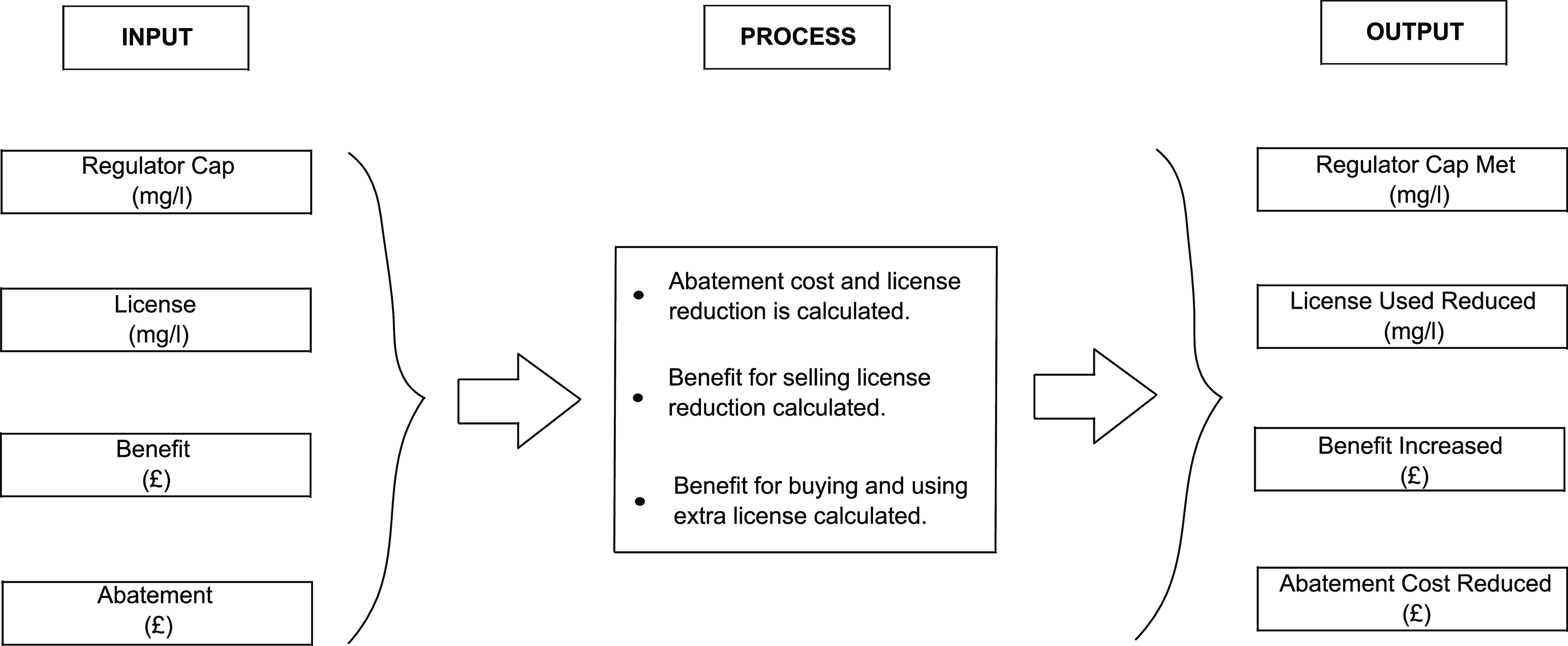
WQT modeling approach. Model inputs, processes, and outputs are introduced.

Figure 1 conceptually shows the elements of the WQT model approach. The input components are shown, and a summary of the processes within the model is detailed. The model outputs are shown where the inputs are either increased or decreased to benefit the water quality and the farm.

### WQT Model Building

The first part of WQT model building is the calculation of the nitrogen load for each farm based on their land use. This nitrogen load is based on the nitrogen input to a crop and the subsequent nitrogen surplus. The nitrogen load contribution for each farm is a calculation based on the average crop yield and average nitrogen input taken from Agriculture in the United Kingdom datasets (2022) and The British Survey of Fertiliser Practice (2021). It is assumed that the yield and nitrogen input rate of each farm are the same based on the data used. To achieve a reduction in nitrogen water pollution, policymakers impose a limit on the nitrogen loading contribution from each polluter (farm) of the water, which is equivalent to a cap.

The second part of the model consists of the costs incurred by the farms for participating in nitrogen water pollution trading. In this WQT model, costs are associated with buying pollution and investing in pollution reduction measures (abatement). The nitrogen WQT model simulates the most cost-effective scenario for the whole catchment area to reduce nitrogen water pollution.

The third aspect of the nitrogen WQT model is the net benefit achieved by the whole catchment. Two scenarios are simulated in this paper, namely the no trading scenario with a cap and the trading scenario with a cap. The no-trading scenario will show how the farms will respond and adhere to the application of a nitrogen water pollution license. This would include the individual costs associated with meeting the cap. The trading scenario will show how the farms within the catchment can work together to lower their overall nitrogen load contribution costs effectively and increase their net benefit. Farms that can invest in abatement at a lower cost do so, which consequently generates extra nitrogen water pollution allowances that can be sold to other farms that find it cheaper to buy nitrogen pollution.

The fourth part of the model is the application of constraints designed to ensure the nitrogen water pollution license assigned to each farm is respected. The total nitrogen water pollution used, bought, and sold by the farms needs to be less than or equal to the cap assigned at the gauge for the crop growing period. In addition, the nitrogen water pollution license that is sold by the farms has to be equal to the nitrogen water pollution license that is bought by the farms.

### WQT Model Assumptions

In building the WQT model, we assumed the following: The first assumption of the nitrogen WQT model is that the pre-trade nitrogen load contribution calculations are based on the farmer’s productivity at the optimal level. The second assumption is that this is a market where license holders are willing to participate in trades, and the price of nitrogen water pollution licenses is known to all. The third assumption is that the administration, legal, and monitoring costs associated with transactions are the same for each license holder that wishes to trade. The fourth assumption is that the crops grown by each farm and their area are the same throughout the crop growing period, and there is no shifting between crops outside those mentioned in this paper.

### Nitrogen Load from Crops

The total nitrogen water concentration is based on all the polluting sources upstream of the gauge in addition to the natural nitrogen. The nitrogen load is the nitrogen that reaches a water body from nitrogen surplus (nitrogen input minus nitrogen uptake) on the field; the pathway of focus in this paper is surface runoff.

The nitrogen inputs on a cropland are as follows: fertilizer (*N*_fer_), manure (*N*_man_), biological fixation (*N*_fix_), and atmospheric deposition (*N*_dep_). Data on the nitrogen inputs for each crop was taken from The British Survey of Fertiliser Practice (2021). The NUE of a crop is the ratio of the crop nitrogen uptake to the total nitrogen input.^6^ The nitrogen yield of the crop is calculated based on the nitrogen uptake of a crop minus the nitrogen residue. Data for the nitrogen yield of each crop was taken from Agriculture in the UK datasets (2022).

The NUE was calculated by
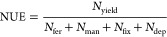
1

The *N*_sur_ of a crop is the excess nitrogen left in the soil after nitrogen is applied and taken up by the crop. The *N*_sur_ was calculated by
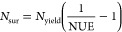
2

Models designed to calculate the nitrogen load for UK specific regions exist, such as HYPE^36^ and INCA,^37^ although these will provide more accurate nitrogen load values, we chose life cycle assessment (LCA) methodologies to estimate the quantity of nutrients reaching rivers.^38^ This is because globally standardized LCA solutions are becoming increasingly used both in research and by corporations to estimate environmental impacts. The fate factor (FF) developed by Jwaideh, Sutanudjaja, and Dalin (2022) provides a globally standard FF that fits within LCA solutions and is based upon a global nutrient model—an integrated model to assess the global environment—a global nutrient model. This method incorporates various distinct local characteristics such as slope, landcover, texture, temperature, soil loss, precipitation, soil drainage, and soil organic carbon, among many others, at a 5 arcmin resolution. Although using a country specific nutrient model (e.g., U.K. specific) would potentially be more accurate for the U.K. It would not produce comparable results to other countries and be in line with LCA methodologies which recommend globally standardized methods.

The FF, as termed by the research, considers the complexities of soil with the factors mentioned above. The current research uses FF in order to explicitly assess the emission of nitrogen through the application of fertilizers. This method has been chosen as the land type has been classified clearly in the FF research conduction by Jwaideh, Sutanudjaja, and Dalin (2022), where they have categorized the land types as arable, grassland, and natural land. Additionally, the FF research also looks into the transport model through which nutrients are delivered to water bodies such as slopes and drainage, which largely affect the transport model
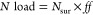
3

To calculate the added concentration of nitrogen in the river from each farm, we used the mass of nitrogen load (mg) and the volume (liters) of the river flow over one crop growing period. The crop growing period differentiates for each farm. Fertilizer is added to the soil when a crop is sown; the largest application of fertilizer is made at the peak of the crop’s growing cycle, when the plant is leafing out.^39^ As each farm grows different crops, and it is uncertain to know exactly what month the peak application of fertilizer occurs, we used the average monthly river flow from the years 2014–2019^40^ to represent the month where fertilizer is applied in its highest quantity by the farms in any particular crop growing cycle. The following calculation was used
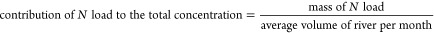
4

Equation 4 was used to calculate the *N* load from each farm identified above the gauge. As this area of Suffolk has high agricultural coverage and a low human population density,^41^ in this paper, we assumed that most of the total nitrogen water concentration is fairly constant and increases in nitrogen levels come from farms (non-point source).

### Market-Based Model Building

The paper presents a pairwise-based trade where two parties buy and sell nitrogen water pollution to each other. Below, we demonstrate how the model is built, with the mathematical formulations explained at each step. The pathways for buying and selling nitrogen water pollution are further explained in Figure 3a.

### Introducing Abatement Measures

Nitrogen pollution abatement measures are used in farming to reduce nitrogen loading. Several abatement measures exist, including changes in agricultural practices and adopting measures such as buffer strips and wetlands that filter runoff,^18^ each differing in cost and effectiveness.

### Costs

In order to take part in trading, the buying and selling of nitrogen water pollution between two parties must occur. Both scenarios come with an associated economic cost, however, the WQT model will simulate the most cost-effective scenario for the overall catchment.
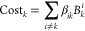
5where Cost_*k*_ represents the economic cost incurred by the farm, which can be from buying extra nitrogen water pollution or investing in abatement. β represents the economic cost, β_*ik*_ is the economic cost farm *k* pays to farm *i*. *B* is the unit of pollution bought, and *B*_*k*_^*i*^ is the unit of pollution farm *k* buys from *i*. Therefore, the cost incurred by a farm is the economic cost multiplied by the unit of pollution bought or reduced.

### Benefit

A farm earns its economic benefit by using its nitrogen water pollution license though growing crops. A farm can also buy extra nitrogen water pollution; this will allow the farm to use more nitrogen and increase its yield and, therefore, its economic benefit.
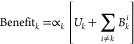
6

The Benefit_*k*_ is a monetary value and is how much economic benefit the farm (*k*) makes. It is what the farm (*k*) uses from its own nitrogen water pollution license, represented as *U*_*k*_, plus the unit of pollution farm *k* buys from *i*, represented as *B*_*k*_^*i*^. The unit of pollution used is multiplied by the economic value represented as ∝_*k*_.

### Selling Benefit

The selling benefit is a monetary value and is the economic benefit gained from a selling nitrogen water pollution license. The selling benefit is represented as *BS*_*i*_ and is calculated by how much money *I* receive from *k*. β_*ik*_ represents the economic cost farm *k* pays to farm *i* for a unit of pollution *i* sold to *k*, represented as *B*_*k*_^*i*^. Equations 5 and 7 are based on the same concept of buying and selling and therefore use the same symbols.
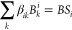
7

### Net Benefit

The net benefit is the overall benefit gained by the farm after buying and selling nitrogen water pollution licenses. The net benefit is designated as the objective function and is calculated below
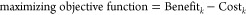
8

The objective function quantifies the economic net benefit generated from buying and selling nitrogen water pollution.^42^ The model is solved by maximizing the objective function, where the benefit for each participant is quantified by how much each farm uses from its own nitrogen water pollution license and how much nitrogen water pollution the farm buys or sells.

In the objective function, *k* represents the owner of the pollution. The objective function calculates how much benefit farm *k* makes minus the cost farm *k* incurs for participating in trading.

### Model Constraints

Nitrogen water levels measured in mg/L are regularly regulated in the U.K. When nitrogen water levels are found to be above desired levels, water quality regulators apply nutrient control measures such as a limit on nitrogen loads entering the water body.^43^ The term regulator is defined as quantifiable constraints on *N* consumption, production, or loss.^44^ Such constraints limit the nitrogen load from polluters within the catchment. A maximum nitrogen load limit is set as a cap, this cap corresponds to the environmental regulator’s water quality target.^12^ To demonstrate how the model works, the regulator in eq 9 sets a cap that totals the sum of the nitrogen water pollution licenses assigned.

The first constraint introduced is the nitrogen water pollution cap imposed by the regulator. We set the regulator cap at the gauge of the waterbody. The equation below ensures that the nitrogen water pollution at each time step is equal to the amount traded in the previous timestep
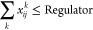
9

The total nitrogen water pollution produced by the farms (*x*) must be less than or equal to the regulator’s cap. *k* represents the owner of the nitrogen water pollution, *i* is the seller, and *j* is the buyer. Equation 9 ensures the nitrogen water pollution *i* sells to *j* and, subsequently, the nitrogen water pollution *j* buys from *i* is less than or equal to the regulatory imposed cap.

Each farm is assigned a nitrogen water pollution license that they cannot exceed. Therefore, the nitrogen water pollution each farm uses and sells is less than or equal to their license.
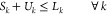
10Here *S*_*k*_ represents what farm *k* sells, *U*_*k*_ represents what farm *k* uses from its own nitrogen water pollution license, and *L*_*k*_ is the farm’s license limit.

A further constraint is designed to ensure the nitrogen water pollution sold by the farms is equal to or less than their nitrogen water pollution license; the equation below is enforced.
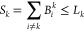
11

In the above equation, *S*_*k*_ represents what farm *k* sells, and what farm *k* sells to farm *i* (*B*_*i*_^*k*^) must be less than or equal to its license *L*_*k*_.

The total nitrogen water pollution for each farm after trading is *P*_*k*_.
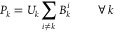
12

In the above, *P*_*k*_ represents the total nitrogen water pollution used by the farm *k*. *P*_*k*_ is equal to *U*_*k*_, which is how much nitrogen water pollution farm *k* uses from its own license, plus *B*_*k*_^*i*^, which is how much extra nitrogen water pollution farm *k* buys from *i*.

Therefore, following on from eq 12, the value of *P*_*k*_ which is the total nitrogen water pollution used by the farm, must be less than or equal to what *i* sells to *j* and what *j* buys from *i*.
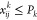
13

### Mass Balance

A trading balance is ensured by the application of the mass balance equation. The total pollution sold in the catchment is represented as *x*, and the owner of the pollution is represented by *k*. Therefore, the nitrogen water pollution *i* sells to *j* is equal to the pollution bought by *j* from *i*.
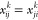
14

## Case Study Application of WQT: UK Suffolk Region

This section introduces the case study river and outlines the further constraint equations added to the model to reflect a local, real-life scenario. For the application of the model to the UK, Nitrogen Vulnerable Zones (NVZ) were identified, as shown in Supporting Information Figure 1. In 2022, Defra designated 55% of England as NVZ, most of which was located on the east coast.^46−49^ In this case study, the advisor and regulator are the Environmental Agency (EA). As suggested by EA’s database,^53^ the River Alde, based in Suffolk, has high levels of nitrogen in its water bodies, where agriculture is the predominant land use.^45^ As this area has a low human population density,^41^ we assume that the natural water nitrogen concentration is fairly constant and that any increases observed come from farming activity. Data for the total nitrogen surface water levels in the River Alde (2014–2019) are shown in Supporting Information Table 2.

**Figure 2:**
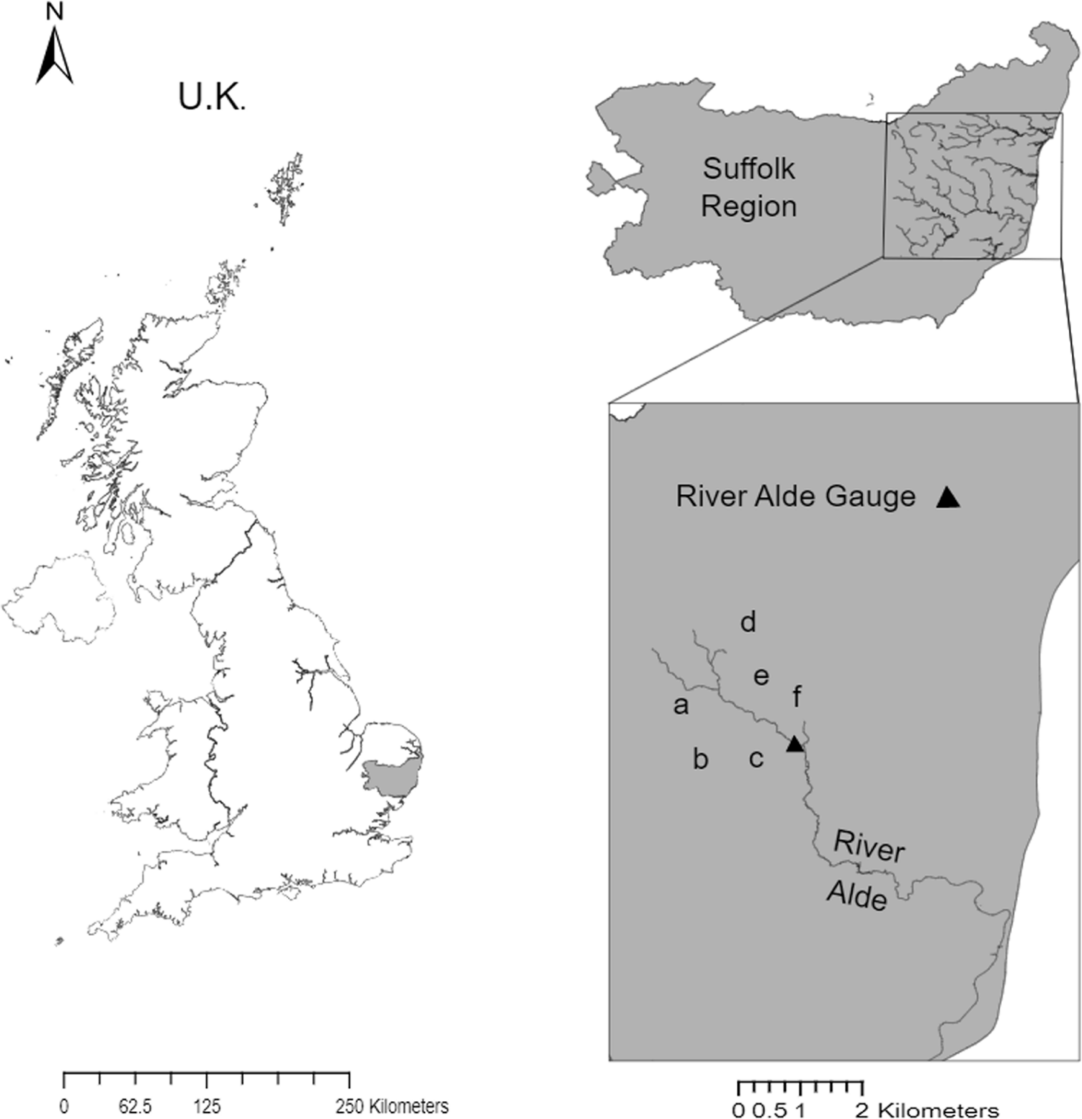
Case study area. UK map showing the location of the Suffolk region, a map of the River Alde gauge, and the approximate location of the six farms a–f.

Figure 2 shows a map of the UK with the Suffolk region identified. The Suffolk region is focused on showing the location of The River Alde, with a further focus on the approximate locations of the six farms within the gauge.

### Model Application to The River Alde

The River Alde has a length of 22 km and is next to the coastal waters of the North Sea. This area is designated as an NVZ, meaning that total nitrogen water levels contain or could contain nitrate concentrations above 50 mg/L, and the area is eutrophic or could become eutrophic if preventative action is not taken.^47−49^ At present, there is currently no nitrogen WQT occurring within this catchment.

Despite its increasing agriculture activity, there has been very little attention received in this part of the UK;^45^ therefore, applying a nitrogen WQT model to this coastal Suffolk river is worth exploring and, in addition, will aid in providing ecological significance with respect to freshwater and coastal habitats.

We applied WQT to six local farms located above the River Alde gauge; the approximate locations of the farms to the gauge are shown in Figure 2. It is noted that the number of farms used in this paper is a small sample size and is therefore not a full representation of all the farms within the River Alde catchment. This number of farms is, however, sufficient to prove the concept of the model and simulate WQT in the River Alde catchment. The farms were each contacted by telephone, and data on the size of the farms and the crops grown was collected (see Table 1). Because of data protection, the names of the farms are not used; instead, they are renamed as farm a–f.

**Table 1:** Size of Farms and Crops Grown^a^

farm	hectares	crops/ha
a	121	wheat—81
		rapeseed—20
		barley—20
b	80	wheat—40
		barley—40
c	80	sugar beet—80
d	101	wheat—51
		rapeseed—25
		barley—25
e	60	wheat—30
		rapeseed—15
		barley—15
f	101	wheat—61
		rapeseed—40

a The size of the farms and the crops grown are shown. The average size of the farms in this case study is 94 ha; we note that this is below the average farm size in our region of interest, which is 121 ha. Based on our farming data, we find that cereals are the crop most commonly grown in this area (wheat and barley), with rapeseed and sugar beet following behind. This is in accordance with the Total Income from Farming (2020) report that details key statistics for farming in the East of England, which states that cereals are the crops mostly grown in this area.

### Nitrogen Load Contribution

Agriculture is a non-point source of pollution. Each farm contributes a nitrogen load to the river catchment. The nitrogen load contribution is based on the crops the farm grows, the size of the farm, and their proximity to the river. The nitrogen load for the crops grown was calculated based on the nitrogen yield per hectare^34,48^ and nitrogen input per hectare.^35^ The NUE for each crop and the nitrogen surplus per hectare was calculated using eqs 1–3. The nitrogen surplus per hectare was multiplied by the FFs taken from Jwaideh, Sutanudjaja, and Dalin, (2022) as shown in Supporting Information Table 1.

We found that the EA measures total nitrogen water levels in mg/L,^50^ as we want to demonstrate nitrogen water pollution trading, we calculated the nitrogen load contribution to the total water nitrogen concentration for each farm using eq 4. Data for the river flow was taken from the National River Flow Archive (2022) and is shown in Supporting Information Tables 2 and 3.

Fertilizer application depends on the crop growth cycle; although a small amount of fertilizer is applied when seeding, the largest amount of fertilizer is applied at the peak of the crop growth cycle. Because our data consists of different crops per farm, the timings of the nitrogen application peak is different for each crop and farm. As we were unable to determine exactly when the largest volume of nitrogen application is added to each crop, we used the monthly average river flow for the River Alde. The nitrogen load was divided by the average monthly river flow; this gave us the average nitrogen load contribution for one month (eq 4).

The National River Flow Archive (2022) provides the gauged daily flow data; it presents the data as the mean river flow in cubic meters per second (m^3^/s). We worked out the average daily river flow for each month in m^3^/s). To work out the average monthly river flow, we multiplied the average daily river flow for each month by the number of seconds in a day (86,400) and multiplies by the number of days in the corresponding month. The final value was multiplied by 1000 to convert m^3^/s to month per liters. The average monthly river flow in liters per month was calculated to be 832,292,661.6 L/month, as detailed in Supporting Information Tables 3–6.

Equation 4 was used to calculate the nitrogen load contribution for each crop (Supporting Information Table 1); the nitrogen load contribution was summed for each farm, as shown in Table 2. This nitrogen load contribution was multiplied for each crop according to the number of hectares it is grown. Data from Table 1 and Supporting Information Table 1 were used to produce the total nitrogen load contribution for each farm, as shown in the last column of Table 2.

**Table 2:** Nitrogen Load Contribution per Crop and per Farm^a^

farm	wheat (mg/L) (×10^–^^4^)	barley (mg/L) (×10^–^^4^)	rapeseed (mg/L) (×10^–^^4^)	sugar beet (mg/L) (×10^–^^5^)	total nitrogen load (mg/L) (×10^–^^3^)
a	10	2	2	0	1
b	5	4	0	0	0.8
c	0	0	0	9	0.1
d	6	2	3	0	1
e	4	1	2	0	0.7
f	7	0	5	0	1

a The values for each farm show the nitrogen load contribution per crop before trading. This value was multiplied by the number of hectares it was grown on; for example, farm a grows wheat on 81 ha (see Table 1), so 10 × 10^–4^ was multiplied by 81 to calculate its nitrogen load contribution for wheat. This was subsequently done for barley and rapeseed; the values were summed, and this was used as the total nitrogen load for the farm, as shown in the last column of Table 2.

### Cover Crops as the Abatement Measure

Abatement measures are used by farmers to reduce their nitrogen loading contributions. Data for the abatement measures used by each farm was not available. Instead, the top abatement measure from the Catchment Sensitive Farming (CSF) (2019) report was used, which was cover crops.^49,51^ Cover crops are grown as a non-cash-profit crop and are planted for the purpose of protecting soil and retaining nitrogen in the field; they reduce the nitrogen load contribution of a farm by 30%.^52^ Therefore, if a farm invests in the abatement measure of cover crops, it reduces its nitrogen load contribution by 30% in one crop growing period. The cost of the abatement measure per hectare is £124.^53,54^ WQT is premised on the assumption that farmers that can invest in abatement at a lower cost will do so and hence reduce their nitrogen loading contribution; it is therefore assumed that not all farms will invest in abatement. Although all farmers were assigned the same abatement measure in this case study, the cost differed because the sizes of the farms were different and the crops grown varied.

### Application of the Regulator Cap and Nitrogen Water Pollution License

When waterbodies are found to have high levels of pollutants, such as nitrogen, governing bodies and local authorities impose pollution restrictions on the users of the water. The restrictions are imposed to limit (cap) the amount of pollution emitted by the users of the water, such as the enforcement of a nitrogen water pollution cap at the gauge. The cap is then distributed to users of the water, and this becomes their assigned pollution license, which they cannot exceed. Users of the water, such as farmers, must reduce their nitrogen loading contribution, and this can be costly. Abatement measures differ in cost and efficiency and are tailored to each farm. In this paper, we impose a nitrogen water pollution cap at the gauge, and the users of the water (farms) are consequently assigned a nitrogen water pollution license based on this cap.

In this case study, we used the EA as the advisor and regulator. The application of the regulator cap was centered around the abatement measure applied to this case study. Planting cover crops reduces farmers nitrogen load by 30%;^49^ therefore, assigning a cap based on a 30% reduction of nitrogen load would see fit. This consequently meant that the farmers were assigned a nitrogen water pollution license that was 30% less than their nitrogen load contributions.

**Figure 3:**
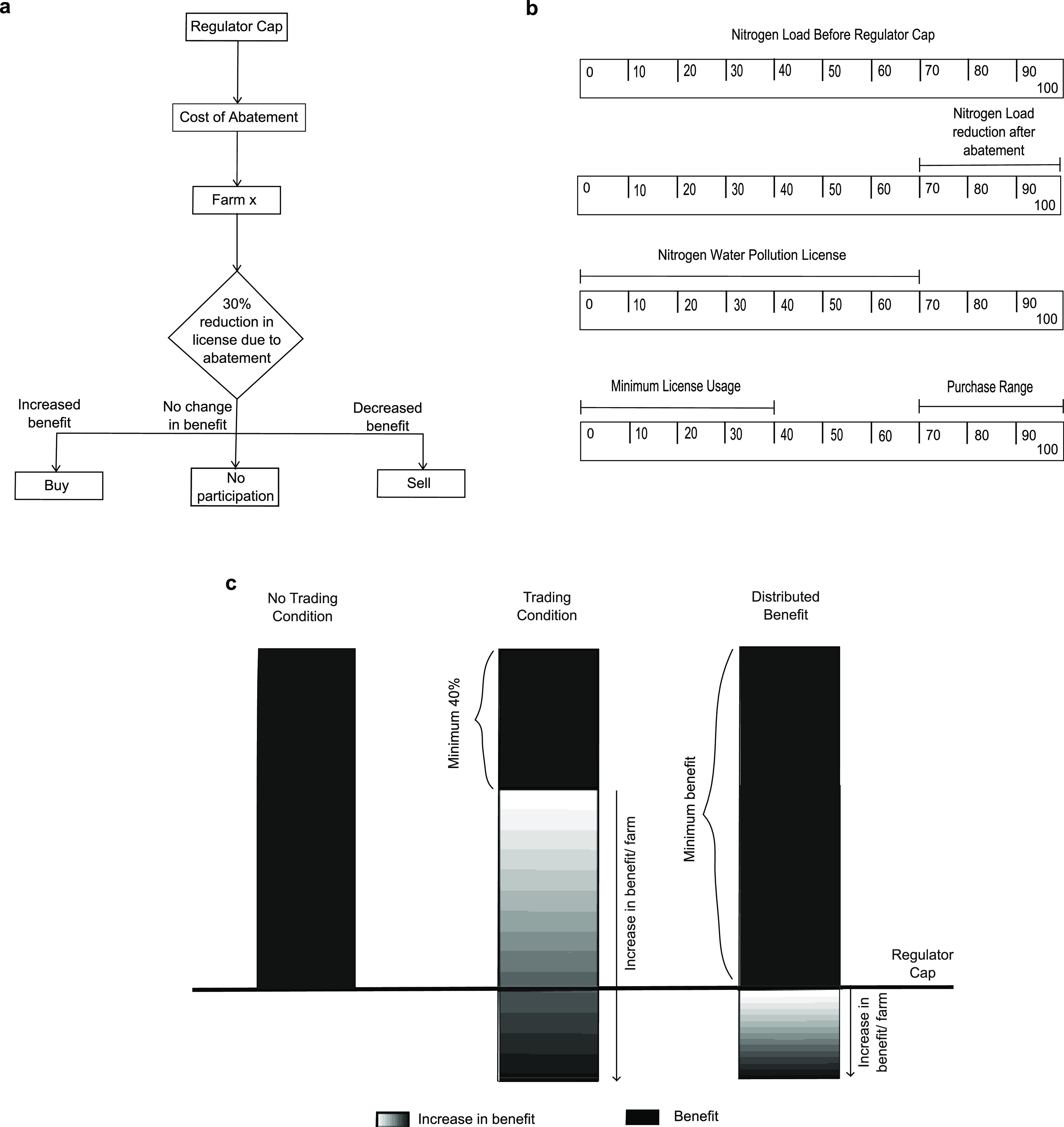
Buying and selling pathways and application of cap and benefit outcomes. (a) Pathways for buying, no participation, and selling nitrogen water pollution. (b) Assigning an EA regulator cap and nitrogen water pollution license to each farm. (c) Benefit for no trading condition, minimum benefit, and increase potential for trading condition and distributed benefit.

Figure 3a shows the three pathways that farmers can take when nitrogen WQT is applied to a catchment. The farms all have the option to invest in abatement; however, depending on their pollution reductions and benefit outcomes, their pathways will be different. Figure 3b demonstrates how an EA cap is set and how each farm is assigned a nitrogen water pollution license based on this cap. The license assigned is 30% less than their nitrogen load; therefore, in order for farms to reach their maximum yield, they can buy up to the 30% reduction back from another farm as detailed in eq 15. Depending on the pollution reduction levels, cost associated with abatement, benefit for selling, benefit for using all their licenses, and benefit for buying pollution, the farms can trade with each other to achieve the best environmentally friendly status while increasing their benefit. The farms, however, must not exceed the regulator cap set at the gauge. Figure 3c demonstrates the benefit when not trading, where farms will use all of their license, the benefit when trading, where farms have to use a minimum of 40% of their license, as detailed in eq 16; and the benefit distribution, where the benefit for using all the license is met, and an increase in benefit from trading is shown in Table 4.

### Transaction Cost

The transaction cost in this model reflects the abatement cost, which is £124 per hectare. The transaction cost is calculated using eq 5. There are two scenarios for the transaction cost. The first scenario is the transaction cost for reducing nitrogen water pollution, it is the cost of abatement multiplied by the nitrogen water pollution reduced. The second scenario is the transaction cost associated with buying nitrogen water pollution, it is the cost of abatement multiplied by the nitrogen water pollution bought.

In this case study, it is assumed that the legal and monitoring costs for all farms are the same and included in the transaction cost. The trading ratio is a 1:1 trading ratio, where the pollution reduced is equal to the pollution sold.

### Farm Gate Price

The farm gate price (GDP) is what the farmer receives for its products after all the input costs; it is the benefit that the farm earns for selling its crops, as detailed in eq 6. The farm gate price for each crop was taken from the API—Index of the prices of agricultural outputs and inputs—statistics notice (data to June 2022) and is detailed in Supporting Information Table 6.

### Modeling License Restrictions

We set the limit on the nitrogen water pollution each farm could buy at 42% of their nitrogen water pollution license. Therefore, the maximum nitrogen water pollution a farm can buy during trading is equivalent to their nitrogen load.
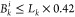
15*B*_*k*_^*i*^ is what farm *i* buys from farm *k*, and this value has to be less than or equal to the license (*L*_*k*_) multiplied by 0.42.

We were conscious that during trading, farmers can shift crop production to more economically beneficial crops, and if they wanted to sell all their nitrogen pollution licenses, they could. To ensure farmers continue business/productivity as usual, or at least to a certain extent, we added a further constraint where farmers must use 57% of their license. This 57% was assigned to reflect literature stating that crops only take up 30–40% of the applied nitrogen. 57% of the nitrogen water pollution license is equal to 40% of the nitrogen load. Therefore, this 57% minimum use of nitrogen in water pollution license would be enough to satisfy crop nitrogen requirements, at least in theory.
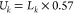
16

To sum up the license restrictions applied to the WQT model, farmers have to use a minimum of 57% of their license and can buy a maximum of 42% of their license.

## Results and Analysis

### Results Summary

The nitrogen load for six farms upstream of the River Alde gauge was calculated based on the crops grown. Each farm was assigned a nitrogen water pollution allowance based on 70% of their nitrogen load; this was summed and set as the gauge EA regulator cap (0.0037 mg/L). The data was entered into the model to simulate how the farms would respond. Two scenarios were simulated with the model: no trading and trading.

**Figure 4:**
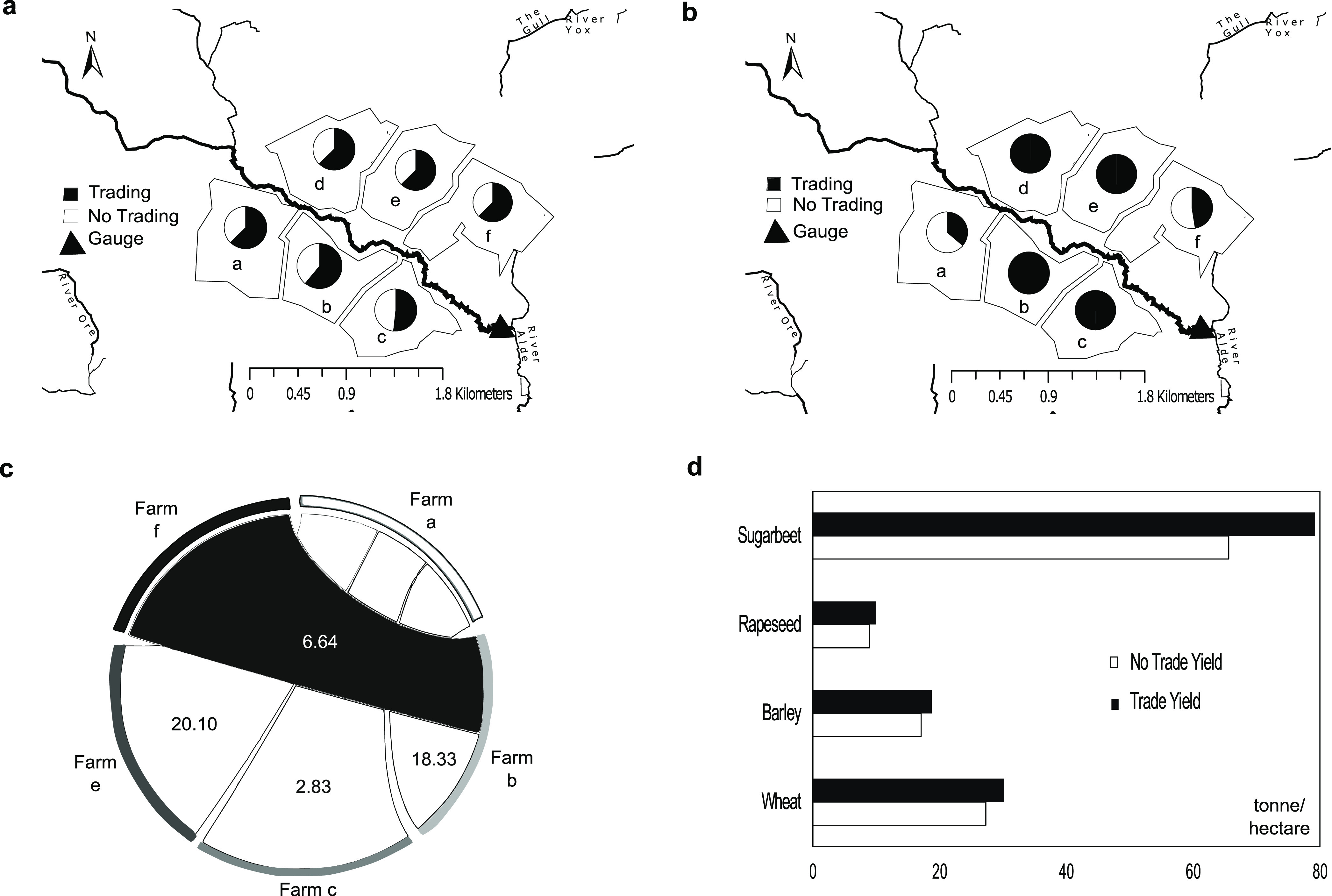
WQT modeling output. (a) Visual representation of benefit (GDP): no trading and trading. The pie chart represents the benefit when trading; we can see the no trading benefit as a lower percentage of the benefit when trading. (b) Visual representation of license used (mg/L): no trading and trading. The pie chart equals the license used when not trading; we can see the percentage of license used when trading. (c) Nitrogen water pollution is measured in mg/L. The ribbon attached to the segment represents the buyer of the pollution, and where the ribbon ends before the segment, it indicates the seller of the pollution. The license bought is represented by the value inside the ribbon, which is ×10^–5^. In this crop-growing period, farms b, c, and e bought nitrogen and water pollution from farms a and f. Farm d did not participate in trading. (d) Crop yield changes (tonnes) for the six farms with no trading and trading.

Figure 4a shows the visual representation of the farms near The River Alde and the benefits before and after trading. The pie chart equals the benefit when trading and demonstrates the percentage of benefit gained when not trading. We find that the benefit when not trading is lower than when trading. Figure 4b shows the license used when trading. The pie chart is equivalent to the license used when not trading (100% of license). We can see that only farms a and f reduce their license when trading, and hence these are the farms that sell their license (Figure 3c). Figure 4d shows the total crop yield changes for the crops grown by the farms in this case study. Of the four crops grown by the farms in this case study, it can be seen that during trading, the crop yields increased for sugarbeet, barley, and wheat. The rapeseed yield decreases very slightly.

The catchment benefit increased by 22%; however, farms a and f had a decrease in benefit, and farm d had no change. Table 3 shows that the nitrogen water pollution levels were reduced, and therefore it is important that farmers participate. To incentivize all the farmers within the catchment to trade, the benefit was distributed to the farms, as shown in Table 4.

**Table 3:** Comparison of Trading and Not Trading under a Cap^a^

	no trading				trading				
farm	benefit (GBP)	pollution bought (mg/L)	pollution sold (mg/L)	license used (mg/L)	benefit (GBP)	pollution bought (mg/L)	cost of buying pollution (GBP)	pollution sold (mg/L)	license used (mg/L)
a	930	0	0	0.0010	532	0	0	0.0004	0.0006
b	1117	0	0	0.0006	1562	0.0003	33	0	0.0006
c	8282	0	0	0.00007	11,743	0.00003	4	0	0.00007
d	931	0	0	0.0008	931	0	0	0	0.0008
e	931	0	0	0.0005	1304	0.0002	26	0	0.0005
f	941	0	0	0.0008	853	0	0	0.00006	0.0008
total	13,133	0	0	0.0037	16,925	0.0005	63	0.0005	0.0032

a The joint yearly net benefit increases by 22%, and the license used by the farms decreases by 14% compared to the results from not trading. In addition, when trading, the total pollution bought equalled the total pollution sold, indicating that the pollution limit was not exceeded.

**Table 4:** Benefit Distribution for all the Farms when Trading^a^

farm	benefit distribution (GBP)	percentage increase (%)
a	1563	67
b	1749	56
c	8914	7
d	1563	67
e	1563	67
f	1573	67
total	16,925	22

a The benefit was distributed to all the farms to match their benefit before trading. The surplus was further distributed to all the farms, ensuring they all had an increase in benefit and were encouraged to participate in WQT.

The benefit from trading was distributed to all the farms. We can see in Table 3 that farm c had the highest benefit, but once the benefit was distributed, its percentage increase in benefit was the lowest of all the farms (see Table 4).

## Discussion

The increasing usage of nitrogen in agriculture is consequently leading to nitrogen water pollution in the UK, with 55% of the waterbodies in England designated as NVZs (Supporting Information Figure 1). Subsequently, there is a possibility for advisors such as the EA to recommend local regulators impose nitrogen water pollution caps in the near future. As a sustainable solution for maintaining water quality and food production, this study has simulated nitrogen WQT and applied it to six non-point sources of pollution in the River Alde catchment. This is the first investigation of a cap and trade market between farms within this region of the UK. In this case study, we have set a cap at the River Alde gauge, assigned a nitrogen water pollution license to each farm accordingly, and observed the trading patterns.

The results in Table 3 show the no-trading and trading scenarios. A comparison of the two scenarios evidently shows that the total catchment net benefit increased by 22% with trading. Despite this overall increase, it can be seen that not all farms achieved an individual increase in net benefit. Farms a and f experienced a decrease in net benefit with trading, and farm d did not experience a change at all. This combination of results indicates that this WQT model does not force farms to participate and allows a farm’s benefit to decrease if it is beneficial for the whole catchment.

Farm a and farm f were the sellers of nitrogen water pollution (Figure 4c), farm a sold 42% of its license, and farm f sold 11% of its license. Farm a and farm f were assigned the highest nitrogen water pollution license (see Table 2). The results suggest that the two farms with the highest license were able to sell more nitrogen water pollution combined than the other farms. They did, however, do this at an economic loss; farm a’s net benefit decreased by 43%, and farm f’s net benefit decreased by 10%. Table 3 shows the cost farms b, c, and e paid to buy the nitrogen water pollution reductions from farms a and f, and this totaled £63. This cost was found to be less than the abatement cost incurred by farms a and f to reduce their pollution (£487). This evidently states that the cost a farmer incurs for reducing pollution is not the same price they sell their reductions for. To meet the cap, we found that if farmers did not participate in WQT, they would all have to invest in abatement individually to lower their nitrogen pollution. Cover crops cost farmers £124 per hectare; therefore, for the whole catchment, we have calculated this cost to be £744 per hectare. Our results have therefore shown that the abatement cost for the whole catchment has been significantly reduced.

Table 3 shows that farms b and c had the highest benefit before trading. This suggests that the combination of crops the farms grow is more economically valuable; although this is true for farm c, we observe that farms a, d, and e grow the same crops as farm b (wheat and barley). Further analyses of the data indicate that farms a, d, and e also grow other crops (rapeseed), and this was the cause for the reduction in their overall benefit. We found that rapeseed had the highest nitrogen load compared to the other crops (see Table 3). Farm b only grows wheat and barley; therefore, its economic income was higher in relation to its nitrogen load. Farm e bought extra nitrogen for water pollution; it was also the smallest farm and had the lowest nitrogen water pollution license. Despite this, its benefit before trading was the same as farms a and d. It would be fair to suggest that farm e was able to purchase pollution because of its low nitrogen load and hence low nitrogen water pollution license, which was able to produce the same benefit as farms a and d.

The nitrogen WQT model was designed to create a scenario that increases the overall catchment net benefit while simultaneously decreasing the nitrogen loading contribution from the farms. Therefore, it was found that it was most cost-effective for the whole catchment for farms a and f, the farms with the highest license, to invest in abatement and sell their nitrogen water pollution reductions to farms b, c, and e. Although the overall catchment net benefit increased with trading, some farms incurred an economic loss. As a result, to encourage all farms to participate, the money earned from trading was used to match the farmers income before trading. The remainder was divided by the number of farms and evenly distributed (£632), thus increasing all the farms benefits when trading, as shown in Table 4.

Farm d did not participate in trading, meaning that it was most cost effective for it to continue production at a capped rate. Farm c, which grows sugarbeet, was the farm that contributed the highest increase to the overall net benefit. This was because one tonne of sugarbeet was worth £105, and 1 ha can produce 82 tonnes of sugarbeet (Supporting Information Table 7). This was subsequently the most profitable crop in this case study. Sugarbeet was also the crop that had the lowest nitrogen load (Supporting Information Table 1). It is fair to say that if trading restrictions such as those detailed in eqs 15 and 16 were not enforced, we would see farmers shift their production to more profitable crops such as sugarbeet.

We found that when trading, the total economic benefit for one crop growing period for all the farms was £16,925 (see Table 3). This meant that the average value per hectare per year per farm was £2820. When compared to the Total Income from Farming report (2021), the average income per hectare in the East of England in 2020 was £671. Our results estimated a significantly higher value per hectare, and we can assume that this was due to our small sample size based only on six farms. The average size of the farms in this study is 94 ha, which is slightly higher than the UK average of 86 ha; however, it is lower than the average farm size in the east of England, which is 121 ha.

With regard to proving the concept of nitrogen WQT, the model has worked, as the total nitrogen water pollution sold equaled the total nitrogen water pollution bought (0.0005 mg/L). This meant that each farm stayed within their assigned nitrogen water pollution license, and the limit at the gauge was respected. The farms adhered to the constraints imposed; farm a sold 42% of its license, and farm f sold less than 1% of its license; farms b, c, and e bought 42% of their license to reach their nitrogen loading value before trading. The model enabled the farms with the highest nitrogen water pollution license to reduce their nitrogen loading; although the farms experienced a decrease in benefit, this was the most cost-effective scenario. The farms were allowed to sell their nitrogen loading reductions, and this allowed the farms within the catchment to generate a significantly higher net benefit that was distributed equally while remaining within their nitrogen pollution license.

In addition, we observed crop yield changes due to trading under an EA regulator cap. We found that sugarbeet had the biggest increase in yield after trading with an increase of 21%, while wheat had an increase of 1.6%, barley 6%, and rapeseed a decrease of 1% (Figure 4d). The results are positive for the future of food production, as they have shown that crop production can continue at a normal rate and potentially increase, as seen with our crops during the implementation of water pollution caps.

Overall, the WQT model used in the case study has demonstrated the effectiveness of trading in reducing nitrogen pollution. As shown in the case study, when a regulator implements a cap on water pollution, the model works by allowing the farms to meet this cap cost-effectively, reducing the nitrogen loading in the catchment. The model allows farms to reduce their nitrogen usage further where it is beneficial for the whole catchment, generating a higher net benefit with the lowest nitrogen loading levels.

### Limitations

We have designed a WQT model that reduces the cost associated with nitrogen water pollution reduction. When contacting the farms to gather data, we found that farmers were reluctant to provide detailed information about their practices, including abatement measures and costs; only data regarding crops grown and farm size was provided. Because of this absence of data, the cost of the top abatement measure in the UK was used. In addition, the crop growing periods were not given for each crop; we therefore calculated the nitrogen load for each crop based on the average monthly river flow.

We have established that the availability of nitrogen is central to food security. Only one abatement measure (planting cover crops) was incorporated into this case study. Although this was sufficient to simulate how the model works, we need to explore more abatement measures and their effectiveness in reducing nitrogen loads. Adding more abatement measures such as buffer strips, wetlands, and nutrient management/precision farming will make the results more realistic.

This paper only considers one class of non-point source of pollution, meaning that our sample size is small. This is therefore not a full representation of trading within the River Alde; the paper does, however, demonstrate the concept of how the model works. Going forward, this limitation will be overcome by adding a larger number of non-point sources of pollution, including livestock.

### Future Directions

The future directions of this model will be to apply this WQT to a larger case study and include both crops and livestock. In addition, we need to explore new water quality policies and their implications for water quality. We can then create future policy implication simulations, apply them to UK rivers, and observe trading patterns.

For the sustainability of water, our study has demonstrated the value WQT has in the application of a pollution cap at the river gauge. The novelty of this paper lies in the application of a WQT model to non-point sources of pollution; in addition, nitrogen water pollution trading has not yet been considered or explored in UK rivers. We provide a modeling approach that can also be replicated to reflect different settings and include different water-soluble compounds, such as phosphate.

At a larger case study scale, we will be able to observe patterns of different crop productions with trading. More abatement measures will be added to the case study, and the most cost-effective measures will be highlighted. Another important future work direction is how the social and economic conditions of the farm impact how WQT is implemented for nitrogen pollution.

## Conclusions

We have designed a WQT system that simulates trading with non-point sources of pollution. We allocated nitrogen pollution allowances and were able to track the seller of the nitrogen to the buyer. The nitrogen load was assigned an economic value based on the farm gate price. The model was then applied to the River Alde as a case study selected because of its surrounding agriculture activity.

The study provides a robust assessment of the nitrogen and water pollution trades that can occur in one crop growing period under an implemented cap. Unlike existing water trading markets, where trades are designed to benefit the individual, we designed a trading market where farmers can work together to lower their nitrogen water pollution levels jointly, while generating the highest economic income.

We have demonstrated how farmers within a catchment can work together to jointly reduce their nitrogen loading contributions and increase their benefit. Although not all farms experienced an increase in benefit individually, to overcome this, we have matched the no-trading earnings and equally distributed the surplus benefit to all the farms.

Finally, although this trading market is theoretical and the results are model predictions, we find that the nitrogen WQT model provides flexibility to farm owners to buy or sell their nitrogen pollution license, benefiting the environment while simultaneously increasing their land value.

## Supporting Information Available

Description of the model components and further analysis; map of the UK, highlighting the Nitrate Vulnerable Zones in England; data used in the model and the calculations done prior to model inputs; and details of further analysis of data (PDF)

## References

[ref1] FAOSTAT 2014; Food and Agriculture Organization of the United Nations Data http://www.fao.org/faostat/en/#home (accessed July 22, 2017).

[ref2] Lichtenberg E. Some Hard Truths About Agriculture and the Environment. Agric. Resour. Econ. Rev. 2004, 33, 24–33. 10.1017/s106828050000561x.

[ref3] Elmi A.; Madramootoo C.; Egeh M.; Hamel C. Water and Fertilizer Nitrogen Management to Minimize Nitrate Pollution from a Cropped Soil in Southwestern Quebec, Canada. Water, Air, Soil Pollut. 2004, 151, 117–134. 10.1023/b:wate.0000009910.25539.75.

[ref4] Bavar M.; Heidari Sharif Abad H.; Noormohamadi G. The Effects of Different Levels of Nitrogen on Yield and Yield Components of Rainfed Wheat in Two Regions of North Khorasan. Open J. Ecol. 2016, 6, 443–451. 10.4236/oje.2016.67042.

[ref5] Lassaletta L.; Billen G.; Garnier J.; Bouwman L.; Velazquez E.; Mueller N.; Gerber J. Nitrogen use in the global food system: past trends and future trajectories of agronomic performance, pollution, trade, and dietary demand. Environ. Res. Lett. 2016, 11, 095007 10.1088/1748-9326/11/9/095007.

[ref6] Zhang X.; Davidson E.; Mauzerall D.; Searchinger T.; Dumas P.; Shen Y. Managing nitrogen for sustainable development. Nature 2015, 528, 51–59. 10.1038/nature15743. 26595273

[ref7] Greenhalgh S.; Selman M. Comparing Water Quality Trading Programs: What Lessons Are There To Learn?. J. Reg. Anal. Pol. 2012, 42, 105–125.

[ref8] Devlin M.; Barry J.; Painting S.; Best M. Extending the phytoplankton tool kit for the UK Water Framework Directive: indicators of phytoplankton community structure. Hydrobiologia 2009, 633, 151–168. 10.1007/s10750-009-9879-5.

[ref9] Majumdar D. The Blue Baby Syndrome. Resonance 2003, 8, 20–30. 10.1007/bf02840703.

[ref10] Crossman J.; Bussi G.; Whitehead P.; Butterfield D.; Lannergård E.; Futter M. A new, catchment-scale integrated water quality model of phosphorus, dissolved oxygen, biochemical oxygen demand and phytoplankton: Inca-phosphorus ecology (peco). Water 2021, 13, 723 10.3390/w13050723.

[ref11] Hansen L.; Termansen M.; Hasler B. The Potential for Nitrogen Abatement Trading in Agriculture: A Hypothetical Market Experiment. J. Agric. Econ. 2019, 70, 812–839. 10.1111/1477-9552.12319.

[ref12] Hasan S.; Hansen L. B.; Smart J. C. R.; Hasler B.; Termansen M. Tradeable nitrogen abatement practices for diffuse agricultural emissions: A ‘smart market approach. Environ. Resour. Econ. 2022, 82, 29–63. 10.1007/s10640-022-00657-2.

[ref13] Bennett L.; Thorpe S.; Guse A. Cost-effective control of nitrogen loadings in Long Island Sound. Water Resour. Res. 2000, 36, 3711–3720. 10.1029/2000wr900199.

[ref14] Duhon M.; McDonald H.; Kerr S. Nitrogen trading in Lake Taupo: An analysis and evaluation of an Innovative Water Management Policy. SSRN Electron. J. 2015, 57 10.2139/ssrn.2653472.

[ref15] Greenhalgh S.; Selman M. Water Quality Trading, Protecting the Environment, Privately; World Scientific, 2015; pp 335–355.

[ref16] Shortle J. S.; Ollikainen M.; Iho A. Water Quality and Agriculture: Economics and Policy for Nonpoint Source Water Pollution; Palgrave Macmillan: Cham, Switzerland, 2021.

[ref17] Nishizawa E. Effluent trading for water quality management: Concept and application to the Chesapeake Bay Watershed. Mar. Pollut. Bull. 2003, 47, 169–174. 10.1016/s0025-326x(02)00408-3. 12787615

[ref18] Helin J.; Laukkanen M.; Koikkalainen K. Abatement costs for agricultural nitrogen and phosphorus loads: A case study of crop farming in south-western Finland. Agric. Food Sci. 2008, 15, 351 10.2137/145960606780061452.

[ref19] Feng F.; Easter K. W.; Brezonik P. L. Point-Nonpoint Source Water Quality Trading: A Case Study in the Minnesota River Basin. J. Am. Water Resour. Assoc. 2005, 41, 645–657. 10.1111/j.1752-1688.2005.tb03761.x.

[ref20] Fialko K. Three strengths and weaknesses of water quality trading policies, Environmental Finance Blog. 2018, Available at: https://efc.web.unc.edu/2018/04/26/three-strengths-and-weaknesses-of-water-quality-trading-policies/ (accessed June 23, 2023).

[ref21] Finch N. Nutrient Water Quality Trading: A MarketBased Solution to Water Pollution in the Natural State. Ark. Law Rev. 2016, 69, 839–870.

[ref22] Newburn D.; Woodward R. An Ex Post Evaluation of Ohio’s Great Miami Water Quality Trading Program1. J. Am. Water Resour. Assoc. 2011, 48, 156–169. 10.1111/j.1752-1688.2011.00601.x.

[ref23] Prabodanie R.; Raffensperger J.; Milke M. A pollution offset system for trading non-point source water pollution permits. Environ. Resour. Econ. 2009, 45, 499–515. 10.1007/s10640-009-9325-1.

[ref24] Meyer A.; Klein C.; Fünfrocken E.; Kautenburger R.; Beck H. Real-time monitoring of water quality to identify pollution pathways in small and middle scale rivers. Sci. Total Environ. 2019, 651, 2323–2333. 10.1016/j.scitotenv.2018.10.069. 30332665

[ref25] Miller M.; Tesoriero A.; Hood K.; Terziotti S.; Wolock D. Estimating Discharge and Nonpoint Source Nitrate Loading to Streams From Three End-Member Pathways Using High-Frequency Water Quality Data. Water Resour. Res. 2017, 53, 10201–10216. 10.1002/2017wr021654.

[ref26] Bennett J. Markets and government-An evolving balance. In The Evolution of Markets for Water; Bennett J., Ed.; Edward Elgar Publ. Ltd.: Cheltenham, U. K, 2005; pp 1–7.

[ref27] Breetz H.; Fisher-Vanden K.; Jacobs H.; Schary C. Trust and Communication: Mechanisms for Increasing Farmers’ Participation in Water Quality Trading. Land Econ. 2005, 81, 170–190. 10.3368/le.81.2.170.

[ref28] Breetz H.; Fisher-Vanden K. K.; Garzon L.; Jacobs H.; Kroetz K.; Terry R. Water Quality Trading and Offset Initiatives in the U.S.: A Comprehensive Survey. Prepared for the U.S. EPA; National Centre for Environmental Economics: Washington, D.C, 2004.

[ref29] Colby B. G. Transactions costs and efficiency in Western Water Allocation. Am. J. Agric. Econ. 1990, 72, 1184–1192. 10.2307/1242530.

[ref30] Cook A.; Shortle J. Pollutant Trading with Transport Time Lags. Environ. Resour. Econ. 2022, 82, 355–382. 10.1007/s10640-022-00681-2.

[ref31] Kilber V. G. EPA publishes Water Quality Trading Guide. Membr. Technol. 2007, 2007 ((10)), , 6. 10.1016/s0958-2118(07)70217-3.

[ref32] Erfani T.; Erfani R. Fair Resource Allocation using multi-population evolutionary algorithm. Applications of Evolutionary Computation, 2015; pp 214–224.

[ref33] Erfani T.; Mokhtar H.; Erfani R. Self-adaptive agent modelling of wind farm for Energy Capture Optimisation. Energy Syst. 2017, 9, 209–222. 10.1007/s12667-017-0243-y.

[ref34] Agriculture in the United Kingdom data sets. 2022, GOV.UK. Available at: https://www.gov.uk/government/statistical-data-sets/agriculture-in-the-united-kingdom (accessed June 23, 2023).

[ref35] Department for Environment, Food & Rural Affairs. British survey of Fertiliser Practice 2021. 2022, GOV.UK. Available at: https://www.gov.uk/government/statistics/british-survey-of-fertiliser-practice-2021 (accessed June 23, 2023).

[ref36] Lindström G.; Pers C.; Rosberg J.; Strömqvist J.; Arheimer B. Development and testing of the hype (hydrological predictions for the environment) water quality model for different spatial scales. Hydrol. Res. 2010, 41, 295–319. 10.2166/nh.2010.007.

[ref37] Crossman J.; Bussi G.; Whitehead P.; Butterfield D.; Lannergård E.; Futter M. A new, catchment-scale integrated water quality model of phosphorus, dissolved oxygen, biochemical oxygen demand and phytoplankton: Inca-phosphorus ecology (peco). Water 2021, 13, 723 10.3390/w13050723.

[ref38] Jwaideh M. A.; Sutanudjaja E. H.; Dalin C. Global impacts of nitrogen and phosphorus fertiliser use for major crops on aquatic biodiversity. Int. J. Life Cycle Assess. 2022, 27, 1058–1080. 10.1007/s11367-022-02078-1.

[ref39] Jena J. Modern concepts of Fertilizer Application. Advanced Agriculture; New Delhi Publishers, 2020.

[ref40] National River Flow Archive. 2023, National River Flow Archive 2022, Available at: https://nrfa.ceh.ac.uk/ (accessed June 23, 2023).

[ref41] Howden N.; Bowes M.; Clark A.; Humphries N.; Neal C. Water Quality, Nutrients and the European union’s Water Framework Directive in a Lowland Agricultural Region: Suffolk, south-east England. Sci. Total Environ. 2009, 407, 2966–2979. 10.1016/j.scitotenv.2008.12.040. 19217145

[ref42] Erfani T.; Erfani R. An evolutionary approach to solve a system of multiple interrelated agent problems. Appl. Soft Comput. 2015, 37, 40–47. 10.1016/j.asoc.2015.07.049.

[ref43] Stephenson K.; Shabman L. Nutrient assimilation services for water quality credit trading programs: A comparative analysis with nonpoint source credits. Coast. Manag. 2016, 45, 24–43. 10.1080/08920753.2017.1237240.

[ref44] Kanter D. R.; Chodos O.; Nordland O.; Rutigliano M.; Winiwarter W. Gaps and opportunities in nitrogen pollution policies around the world. Nat. Sustain. 2020, 3, 956–963. 10.1038/s41893-020-0577-7.

[ref45] Holden J.; Haygarth P.; Dunn N.; Harris J.; Harris R.; Humble A.; Jenkins A.; MacDonald J.; McGonigle D.; Meacham T.; Orr H.; Pearson P.; Ross M.; Sapiets A.; Benton T. Water quality and UK agriculture: challenges and opportunities. Wiley Interdiscip. Rev.: Water 2017, 4, e1201 10.1002/wat2.1201.

[ref46] Department for Environment, Food & Rural Affairs. Nitrate vulnerable zones. 2021, GOV.UK. Available at: https://www.gov.uk/government/collections/nitrate-vulnerable-zones (accessed June 23, 2023).

[ref47] Environmental Audit Committee–House of commons. UK progress on reducing nitrate pollution. 2021, Available at: https://publications.parliament.uk/pa/cm201719/cmselect/cmenvaud/656/656.pdf (accessed June 23, 2023).

[ref48] Department for Environment, Food & Rural Affairs () Agriculture in the United Kingdom 2021. 2022, GOV.UK. Available at: https://www.gov.uk/government/statistics/agriculture-in-the-united-kingdom-2021 (accessed June 23, 2023).

[ref49] Natural England Publication. Water Quality, Phases 1 to 4 (2006–2018); Natural England Publication, Environment Agency (2019) Catchment Sensitive Farming Evaluation Report, 2019; pp 26–28.

[ref50] Water Quality Data Archive. Open WIMS data. 2022, Available at: https://environment.data.gov.uk/water-quality/view/landing (accessed June 23, 2023).

[ref51] Environment Agency. Catchment Sensitive Farming Evaluation Report–Water Quality, Phases 1 to 4 (2006–2018); Natural England Publication, June 2019.

[ref52] SW6: Winter cover crops. 2015, GOV.UK. Available at: https://www.gov.uk/countryside-stewardship-grants/winter-cover-crops-sw6 (accessed June 23, 2023).

[ref53] Department for Environment, Food & Rural Affairs. Total income from farming for the regions of England. 2022, GOV.UK. Available at: https://www.gov.uk/government/statistics/total-income-from-farming-for-the-regions-of-england (accessed June 23, 2023).

[ref54] Farm business income by type of farm in England 2021/22. 2022, GOV.UK. Available at: https://www.gov.uk/government/statistics/farm-business-income/farm-business-income-by-type-of-farm-in-england-202122 (accessed June 23, 2023).

