# Supplementary material for: Water Quality and Pollution Trading: A Sustainable Solution for Future Food Production
Source: ACS ES T Eng. 2023 Jul 13;3(8):1112–24. doi: 10.1021/acsestengg.2c00383 (PMC10426330; doi:10.1021/acsestengg.2c00383)
Supplement: Supplementary file 1 — ee2c00383_si_001.pdf [file ee2c00383_si_001.pdf]

Supporting Information

Water quality and pollution trading: a sustainable solution for future food production

Jamie Gonzalez Zapata[1], Bharadwaj Vangipuram[1], Carole Dalin[2], Tohid Erfani[1]*

[1]Department of Civil, Environmental and Geomatic Engineering, University College London, London WC1E 6BT, United Kingdom

[2]Institute for Sustainable Resources, Bartlett School of Environment, Energy and Resources, University College London, London WC1H 0NN, United Kingdom

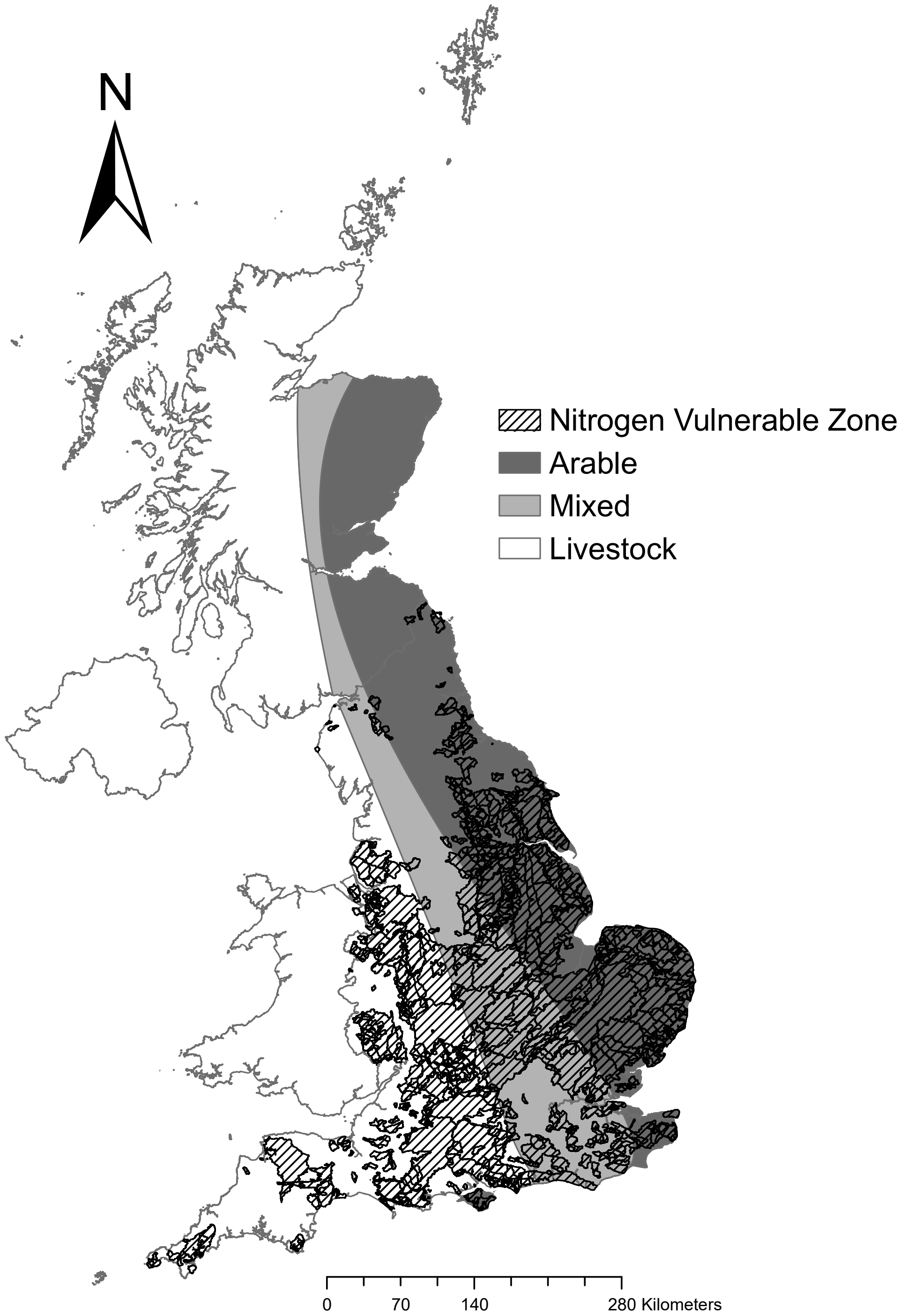

Supplementary Fig 1. UK map with NVZs UK map with Nitrogen Vulnerable Zones 2022 and the farming distribution (Countries (December 2018) Boundaries UK BFC, 2022 & OS Open Rivers - data.gov.uk, 2022).

Table 1. Nitrogen load contribution from each crop for one crop growing period							
**Crop**	**Average N Yield (tonne/ha)**	**Average N input per kg N/ha**	**Nitrogen Use Efficiency**	**N Surplus/ha**	**Fate Factor ($x 10^{-5}$)**	**Nitrogen load (kg/ha)**	**Nitrogen load (mg/ha)**
**Rapeseed**	3.2	197	0.16	194	5.2	0.0100776	10078
**Sugar beet**	82	101	0.81	19	5.2	0.000988	988
**Wheat**	7.8	196	0.04	188	5.2	0.0097864	9786
**Barley**	6.1	151	0.04	145	5.2	0.0075348	7535

Supplementary Table 1 shows the average nitrogen yield of the four crops in this paper, the average nitrogen input, the calculated nitrogen use efficiency (NUE) and the nitrogen surplus. The nitrogen surplus was calculated by the fate factor and this gave the nitrogen load value per hectare. The nitrogen load value was converted from kg to mg per hectare

Table 2. Annual and monthly total nitrogen surface water levels for the River Alde (2014-2019) (mg/L)													
**Year**	**Jan (mg/L)**	**Feb (mg/L)**	**Mar (mg/L)**	**Apr (mg/L)**	**May (mg/L)**	**Jun (mg/L)**	**Jul (mg/L)**	**Aug (mg/L)**	**Sept (mg/L)**	**Oct (mg/L)**	**Nov (mg/L)**	**Dec (mg/L)**	**Yearly average (mg/L)**
**2019**	4	15	13	3	6	2	2	1	2	10	10	13	7
**2018**	13	11	7	6	3	2	2	2	2	2	2	3	5
**2017**	13	12	8	2	2	3	2	2	2	2	2	3	5
**2016**	10	7	8	4	2	5	3	2	3	3	12	9	6
**2015**	7	6	4	3	3	3	2	2	4	3	13	14	5
**2014**	10	7	4	3	13	5	3	6	3	13	11	10	7
**Monthly average**	9	10	7	3	5	3	2	3	3	5	8	9	6

Supplementary Table 2 shows the annual and monthly nitrogen water levels observed in the River Alde for the years (2014-2019) (Open WIMS data, 2022). It can be seen that the nitrogen water levels are the highest in the beginning of the year (Jan-Mar) and towards the end of the year (Nov-Dec). The average nitrogen water levels for the 5-year period is observed to be 6mg/L.

Table 3. River Alde flow m³/s (2014-2019)													
**Year**	**Jan (m³ s)**	**Feb (m³ s)**	**Mar (m³ s)**	**Apr (m³ s)**	**May (m³ s)**	**Jun (m³ s)**	**Jul (m³ s)**	**Aug (m³ s)**	**Sep (m³ s)**	**Oct (m³ s)**	**Nov (m³ s)**	**Dec (m³ s)**	**Yearly average (m³s)**
**2019**	0.06	0.14	0.16	0.05	0.06	0.04	0.04	0.04	0.03	0.11	1.17	1.64	0.3
**2018**	0.88	0.55	1.02	0.13	0.06	0.04	0.04	0.04	0.04	0.04	0.06	0.09	0.25
**2017**	0.32	0.43	0.3	0.07	0.11	0.06	0.05	0.05	0.05	0.06	0.06	0.56	0.18
**2016**	1.17	0.37	0.8	0.3	0.11	0.54	0.1	0.06	0.06	0.06	0.35	0.12	0.34
**2015**	0.66	0.55	0.2	0.13	0.12	0.07	0.08	0.07	0.09	0.08	0.16	0.29	0.21
**2014**	1.02	1.85	0.35	0.13	0.21	0.09	0.08	0.12	0.07	0.23	1.92	1.09	0.60
**Monthly Average (m³s)**	0.7	0.64	0.47	0.14	0.11	0.14	0.1	0.06	0.06	0.1	0.62	0.63	0.3

Supplementary Table 3 shows the River Alde flow from 2014 to 2019. The bottom row and last column show the average monthly river flow and average yearly river flow. It is observed that the monthly river flow is lowest in the summer months of July and August and highest in the winter months November to February. The year with the highest average river flow was 2014 and the year with the lowest river flow was 2017.

Table 4. River Alde flow average 2014-2019		
**Month**	**m^3/s (x 10^{-2})**	**Litres/Month (x 10^7)**
**Jan**	69.1	185.08
**Feb**	65.0	157.23
**Mar**	55.33	148.2
**Apr**	30.0	77.76
**May**	1.28	3.33
**Jun**	14.8	38.45
**Jul**	6.83	18.30
**Aug**	6.83	18.30
**Sep**	6.0	15.55
**Oct**	9.8	26.34
**Nov**	62.5	162
**Dec**	55.33	148.20
**Average**	31.9	83.22

Supplementary Table 4 shows the monthly average river flow from 2014-2019 calculated to be $0.3190655 m^3/s$.

Table 5. River Alde average flow (2014-2019)		
	**m^3/s**	**Litres/ Month**
**Average**	0.3190655	832292661.6

Supplementary Table 5 shows the average river Alde flow in m^3/s converted to Litres/month.

Table 6. Farm gate price of each crop per one tonne

Crop	Farm gate price for one tonne (GBP)
**Wheat**	170
**Barley**	149
**Sugar beet**	105
**Rapeseed**	174

Supplementary Table 6 shows the farm gate price for one tonne for each crop. This was used to calculate the farmgate value for each crop based on their yield per hectare.

Table 7. The farm gate price for the average crop yield		
**Crop**	**Crop yield 2021 (t/ha)**	**Total farm gate price (GBP/ha)**
**Wheat**	7.8	1,326
**Barley**	6.1	909
**Sugar beet**	82	8,282
**Rapeseed**	3.2	557

Supplementary Table 7 shows the average crop yield per hectare and the total farm gate price for one hectare.

Table 8. Farm gate price, nitrogen load and nitrogen water pollution licence							
	**Farm a**	**Farm b**	**Farm c**	**Farm d**	**Farm e**	**Farm f**	**Total**
**Farm gate price (GBP/ha)**	931	1117	8282	931	931	941	13,133
**Nitrogen load ($x10^{-5}$)**	137.57	83.24	9.50	112.87	67.02	120.16	530.35
**Nitrogen water pollution licence ($x10^{-5}$)**	96.3	58.27	6.55	790	46.9	84.11	371.25

Supplementary Table 8 shows the farm gate price for each farm based on the crops they grow. The nitrogen load for each farm is also shown as well as the nitrogen water pollution licence assigned to each farm which is 70% of the nitrogen load.
